# The Revolution of Targeted Therapies in Thyroid Cancer Treatment: Present and Future Promising Anti-Cancer Drugs

**DOI:** 10.3390/ijms26083663

**Published:** 2025-04-12

**Authors:** Sara Gil-Bernabé, Lucía García-DeLaFuente, Ginesa García-Rostán

**Affiliations:** 1 Pathology Department, Faculty of Medicine, Valladolid University, 47003 Valladolid, Spain; 2 Group Pathobiology of Cancer: Inter-, Intra-Tumor Heterogeneity and Molecular Targets, Institute of Molecular Genetics and Biomedicine (IBGM), 47003 Valladolid, Spain

**Keywords:** thyroid, targeted therapies, anti-cancer drug

## Abstract

Thyroid cancer prevalence has increased in the last few decades. Whereas the majority of well-differentiated histotypes have effective therapeutic options, the most advanced cases lacked successful treatment until recent years. Genomic alterations have emerged as targets for new anti-cancer drugs. This molecular knowledge is gradually being translated into sophisticated approaches for the stratification, management, and therapies of patients with thyroid carcinomas. The genomic characterisation of tumours in clinical assistance serves as a tool for enhancing the prognostic assessment of patients with thyroid cancer and predicting their responses to the agents. The MAPK pathway is the most predominantly activated molecular route in this cancer. Several drugs have been developed to inhibit this pathway at different levels. However, the acquired resistance that emerges is the main problem in their use. Other strategies targeting not only driver mutations but also those that confer aggressive behaviour on tumours can be potential targetable options. Due to the new therapies, patients with the most aggressive histotypes have improved survival rates. Adverse events, although manageable, have a high prevalence among the current therapies. Selective inhibitors, immunotherapies, and the combination of both will play a pivotal role in the treatment and the improvements in overall survival in thyroid cancer patients.

## 1. Introduction

Thyroid cancer (TC) is the most common endocrine malignancy, and its incidence has increased in the last few decades [1]. The newly developed drugs have meant a significant milestone in TC. Targeted therapies have been postulated as more effective cancer treatments than previous agents. Discovering genetic biomarkers such as mutations and other genomic events has significantly improved the survival of patients [2].

TC can be categorised based on its cellular origin into follicular-derived and C-cell-derived cancers. Follicular-derived tumours are classified according to their histological degree of de-differentiation. These cancers encompass differentiated thyroid carcinomas (DTCs), high-grade follicular-cell-derived non-anaplastic thyroid carcinomas, and anaplastic thyroid carcinomas (ATCs) [3].

DTC is the most prevalent type of TC worldwide. Approximately 95% of thyroid carcinomas exhibit a well-differentiated pattern. Papillary thyroid cancer (PTC) is the most frequent subtype of DTCs. They are indolent and slow-growing tumours with low metastatic prevalence and excellent prognosis and behaviour. The drivers of this disease affect several genes of the MAPK (Mitogen-Activated Protein Kinase) pathway. The most prevalent is the *BRAF* V600E mutation. Additional drivers include pathogenic variants in *RAS* isoforms and *RET* rearrangements [4]. In recent years, the survival rate for DTCs has significantly improved, largely due to multikinase inhibitors (MKIs).

High-grade follicular-cell-derived non-anaplastic thyroid carcinomas include poorly differentiated carcinomas (PDTCs) and differentiated high-grade thyroid carcinoma (DHGTC). They are entities with a small prevalence (2–15%) and differentiation degree between DTC and ATC. They are fast-growing tumours with the ability to metastasise. BRAF mutations are less prevalent in this histotype, and other mutations appear, such as *TERT*-promoter or *EIF1AX* [5,6].

ATCs represent approximately 1–2% of all TCs. It is a fatal cancer, with a survival of 3–6 months after the diagnosis of fast-growing tumours associated with dysphagia, dyspnea, and distant metastases. Currently, there are few effective treatments available for this disease. The genetic events of these tumours are the previously mentioned, and *TP53* or *PIK3CA* mutations [6,7].

Medullary thyroid carcinoma (MTC) originates from C cells, with approximately 25% of cases linked to familial or hereditary syndromes. The main genetic driver of MTC is *RET* pathogenic variants, followed by *RAS* mutations. The five-year survival rate for MTC is around 65%. The introduction of MKIs and target therapies (selpercatinib) has led to a remarkable improvement in survival outcomes for these patients [8,9].

Advances in understanding the molecular characteristics of TC have significantly improved how we interpret uncertain cytology results and have allowed us to identify distinct subtypes of thyroid carcinoma. Each subtype has unique clinical behaviour, responses to radioactive iodine treatment, and specific targeted therapies. Surgery is the most effective DTC management, followed by radioactive iodine therapy, which improves survival in cases with a risk of recurrence. In advanced TC patients—where the disease has spread and no longer responds to radioactive iodine—molecular testing is used to identify the genetic driver mutations. This information helps select targeted therapies tailored to specific genetic alterations [2,10].

In this review, we seek to gather the most relevant anti-cancer drugs reported in the literature, detailing their advantages and side effects that limit their use. We also approach promising new therapies and future perspectives in TC treatment.

## 2. Genomic Events in Thyroid Carcinogenesis Used as Targets

### 2.1. Driver Mutations

According to The Cancer Genome Atlas (TCGA), the MAPK pathway is activated in 90% of PTC cases. This activation typically results from mutually exclusive mutations in either *BRAF* or *RAS* oncogenes, both serving as key drivers of tumour initiation [11,12]. In some cases, the activation occurs via receptor tyrosine kinase (RTK) gene rearrangements, particularly *RET*, the most common altered gene in MTC [4,13].

**Figure 1:**
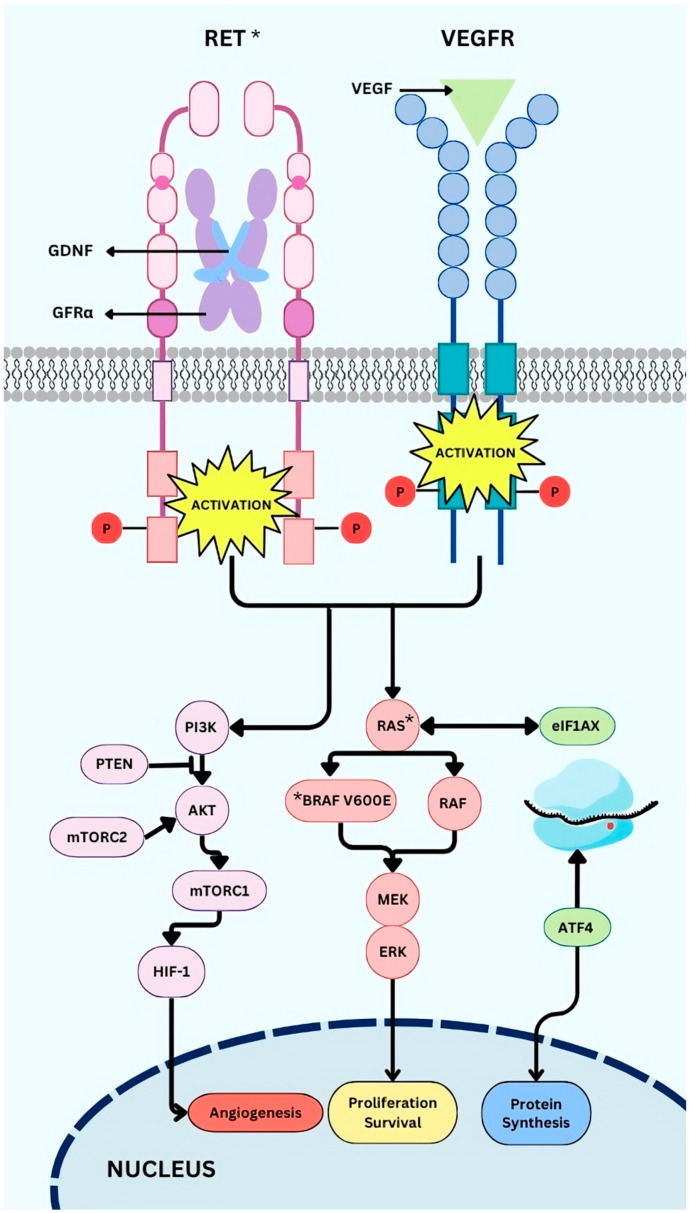
Related thyroid cancer altered pathways. Driver mutations are marked with an * in the figure.

The MAPK cascade is a crucial signalling pathway that regulates cell proliferation, differentiation, survival, and apoptosis. Among MAPK pathways, the RAS/RAF/MEK/ERK route is frequently dysregulated in cancer. RAS activation, triggered by RTK such as EGFR, VEGFR, and PDGFR-β, leads to RAF (including BRAF) activation and stimulates sequentially MEK and ERK. Aberrant activation of this pathway—whether through RAS or RAF mutations, RTK alterations, or ligand-independent mechanisms—drives uncontrolled cell growth, survival, and metastasis [14] (Figure 1).

***BRAF*** is the most common driver mutation in PTC. It is altered in approximately 60% of cases, leading to constitutive activation of the MAPK pathway, which promotes uncontrolled cell de-differentiation, proliferation, and survival [12]. The most prevalent *BRAF* alteration is the *BRAF* V600E mutation, accounting for 47% of cases [15]. Other *BRAF* class mutations—such as *BRAF* K601E and *BRAF* fusions—have been identified, though they are less frequent [11]. Considering other follicular-derived thyroid tumours, *BRAF* mutations have been reported in 19–33% of PDTCs and 19–45% of ATCs [16]. On the other hand, Shi et al. noted that *BRAF* mutations in MTC were an uncommon event. *BRAF* V600E was found in *RET*-negative tumours, with novel mutations (G469A and T599dup), suggesting *BRAF* as a scarce driver of MTC carcinogenesis [15]. The *BRAF* V600E mutation is commonly associated with tumour recurrence, independently of conventional clinicopathologic risk factors [17]. Another remarkable feature of this mutation is that it downregulates the NIS through histone deacetylation, contributing to the loss of radioiodine avidity and treatment failure in PTC [18].

***RAS*** mutations are the second-most prevalent alterations in PTC (35%). *NRAS* is the most common altered isoform, particularly at codon 61, which accounts for 67% of all *RAS* mutations, followed by *HRAS* and *KRAS* mutations [19,20]. These genetic alterations activate the MAPK and PI3K/AKT pathways but with lower signalling intensity than *BRAF* V600E mutations. Consequently, *RAS* mutations are detected across the whole spectrum of follicular-derived thyroid neoplasms, from benign follicular adenomas (20–25%) to 20–40% in PDTC and 10–20% in ATCs [21]. Moreover, *HRAS* and *KRAS* alterations occur in sporadic but not hereditary MTC and rarely coexist with RET mutations [13].

Moreover, *BRAF* and *RAS* are mutually exclusive and are associated with distinct clinical and molecular behaviours [4]. This distinction is partly due to each mutation’s unique MAPK pathway signalling mechanisms. ERK activation in *RAS*-mutant cells induces a negative feedback loop that disrupts RAF dimerisation, reducing pathway output. In contrast, *BRAF* V600E acts as a monomer and bypasses this regulatory loop, resulting in stronger MAPK activation [6].

***RET*** (REarranged during Transfection) is an RTK that activates key signalling pathways like PI3K/AKT, MAPK, and JAK/STAT, driving cell survival and proliferation [22]. *RET* rearrangements lead to the re-expression of their kinase domain, and occur in 10–20% of PTCs, mainly in paediatric and radiation-exposed patients [4,11]. The most common *RET* fusions in PTC are *CCDC6-RET* (RET/PTC1) and *NCOA4-RET* (RET/PTC3), found in 90% of fusion-positive cases, whereas other follicular-derived thyroid cancers rarely harbour *RET* fusions [23].

Attending to MTC patients, *RET* is the main genetic alteration, present in 95% of hereditary cases. MEN2A patients have germline mutations in exons 10/11 affecting the extracellular cysteine domain and causing ligand-independent activation of intra-cellular pathways. MEN2B syndrome is almost exclusively caused by the *RET* M918T mutation (exon 16), which enhances ATP binding and autophosphorylation, leading to dimerisation-independent RET activation. A883F (exon 15) is found in 10% of cases. Somatic *RET* mutations occur in up to 60% of sMTC cases and are associated with tumour aggressiveness, and M918T (40%) is the most common *RET* alteration, along with small deletions and insertions [24,25].

### 2.2. Other Alterations

***TERT***-promoter mutations (C228T and C250T) enhance telomerase expression and contribute to non-telomeric functions, playing a key role in TC progression. They are rare in benign tumours but increasingly prevalent in aggressive subtypes, particularly ATC and PDTC, where they may drive de-differentiation and are closely linked to malignancy [26,27]. Co-occurrence with the *BRAF* V600E mutation amplifies TERT expression by activating ETS transcription factors, which bind to newly created ETS sites in the mutated *TERT* promoter, driving cancer cell immortality [28].

The **VEGF/VEGFR** axis is essential for angiogenesis and tumour vascularisation across various cancers, including TC [6,29]. Under normal conditions, VEGF expression is stimulated by hypoxia and binds to its high-affinity tyrosine kinase receptors VEGFR1 (FLT1), VEGFR2 (KDR), and VEGFR3 (FLT4) [29]. Activating mutations and/or overexpression of these receptors and their growth factors aberrantly activate the PI3K and MAPK pathways. This dysregulation enhances tumour growth and metastasis, making VEGFR a critical therapeutic target in TC treatment [30].

***EIF1AX*** mutations, especially the A113_splice variant, are prevalent in aggressive thyroid cancers like PDTC and ATC and have also been found in benign thyroid nodules [31,32]. These mutations disrupt the proper assembly of the preinitiation complex (PIC), impairing AUG start codon recognition and causing leaky scanning. As a result, translation initiates at downstream start codons, leading to altered protein synthesis [33]. Additionally, *EIF1AX* A113_splice variants activate ATF4 (a key regulator of the stress response), which upregulates survival pathways, supporting tumour progression and cellular adaptation to stress. When co-occurring with *RAS* mutations, they are linked to a poor prognosis as they drive tumourigenesis by stabilising the c-MYC protein and enhancing its activity. This leads to a higher risk of malignancy in thyroid cancer [34]. 

Other alterations could be potential targets of preclinical assays. Other RTKs, such as NTRK or ALK are altered in some types of TC. Alterations in genes such as *PIK3CA* or *TP53* could be a promising treatment in ATCs, the histotype where these mutations are more prevalent [6]. While low-risk and advanced DTCs share common genetic drivers, advanced thyroid cancers acquire additional alterations supporting a tumourigenesis model in which PDTC and ATC evolve from PTC or FTC through the accumulation of key genetic changes [35].

The genetic landscape of primary tumours largely correlates with lymph node metastases. In contrast, distant metastases exhibit greater genetic divergence, including increased alterations in aggressive mutations such as *TERT*-promoter mutations, along with a lower frequency of *BRAF* mutations [26].

## 3. Anti-Cancer Agents in TC

TC has two principal types of inhibitors that have been approved: antiangiogenic MKIs and selectively targeted drugs by the FDA (Food and Drug Administration) and EMA (European Medicines Agency) (Table 1). MKIs with anti-angiogenic properties have shown significant improvements in progression-free survival (PFS) for patients, establishing themselves as a cornerstone in advanced DTC and MTC treatment over the years. In cases of radioiodine-refractory DTC, there have become notable therapeutic options, primarily targeting VEGFR [36]. Selective inhibitors target specific alterations and are used in different histotypes of TC, mainly in the aggressive ones [10].

### 3.1. Multikinase Inhibitors (MKIs)

FDA and EMA have approved all these anti-cancer drugs (Table 1). However, some studies are ongoing due to their side effects and acquired resistance.

**Sorafenib**: This is a kinase inhibitor of VEGFR-1, VEGFR-2, VEGFR-3, RET, BRAF, KIT, and PDGFR [37]. The distal pyridyl ring of Sorafenib directly engages with three amino acids in the ATP adenine binding pocket, while the urea moiety forms multiple hydrogen bonds with the enzyme. The compound seems to stabilise the inactive conformation of BRAF [38]. Sorafenib is suggested as an inhibitor of the growth of RET-driven tumours through a combination of different mechanisms, and it targets both VEGF-dependent tumour angiogenesis and RET-dependent TC proliferative cells [39]. The inhibition of the phosphorylation of initiation factor eIF4E and loss of the anti-apoptotic protein MCL-1 seem to promote the apoptosis of the tumoural cells by Sorafenib [40].

The different phases of its clinical trial called DECISION probed a response rate among radioactive iodine-refractory (RAIR) DTC patients of 12.2% and a PFS of 10.8 months (while the PFS of the placebo cases was 5.8 months). Side effects reported to Sorafenib were secondary malignancy, dyspnoea, and pleural effusion [41]. These have a higher prevalence than other tumours where this drug was tested. Notably, the dermatological problems associated with the treatment in DTCs (e.g., hand–foot skin reaction) increased compared to other cancers [42].

**Lenvatinib**: The targets of Lenvatinib are very similar to those of Sorafenib but also include FGFR. Preclinical trials demonstrated that Lenvatinib suppresses the dissemination of tumour cells by inhibiting lymphangiogenesis and the growth of metastatic lung nodules. This effect occurs through the selective inhibition of VEGFR2 and VEGFR3, which target angiogenesis [43]. Recently, it has been published that Lenvatinib promotes the phosphorylation of ERK1/2 and mTOR, inducing autophagy in TC cell lines [44].

The SELECTION clinical trial showed a PFS of 18.3 months among RAIR patients, opposite to the 3.6 months of placebo cases. The response rate was 64.8% for the Lenvatinib-treated patients. Diarrhoea, hypertension, proteinuria, and decreased appetite were the principal adverse effects [45].

**Vandetanib:** This multikinase targets VEGFR, PDGFR, EGFR, and RET. In recombinant enzyme experiments, Vandetanib demonstrates the inhibition of KDR activity (VEGFR2), the tyrosine kinase of the VEGF-C and -D receptor Flt-4 (VEGFR3) of Flt-1 (VEGFR1). In *RET*-mutant cancers, Vandetinib acts as an anti-angiogenetic and an antineoplastic drug [46]. The structure aligns with a kinase binding motif, where the quinazoline ring occupies the adenine binding site of the kinase. At the same time, the aniline moiety of the molecule inserts into a hydrophobic pocket. This pocket exhibits structural variability across different kinases, contributing to selectivity [47]. The administration of Vandetanib has been approved for MTC patients. The response rate of Phase III of the clinical trial was 45%. Interestingly, the predicted PFS was 30.5 months vs. 19.3 in the placebo group. The side effects reported were diarrhoea, rash, nausea, hypertension, and headache [48].

**Cabozantinib:** This inhibitor targets different kinases, such as MET, RET, KIT and VEGFR. MET is the hepatocyte growth factor (HGF) receptor and its expression is dysregulated in several tumours. HGF is a potent angiogenic factor and acts with VEGF to induce angiogenesis [49]. Data demonstrated that inhibiting MET and VEGFR2 with Cabozantinib blocks the development of MET resistance in other drugs that only target VEGF. Inactivation of both pathways provided an enormous anti-tumour effect through the blockage of downstream phosphorylations [50]. This MKI has been approved for MTC and RAIR DTC in the clinical trials EXAM and COSMIC-311, respectively. In MTCs, the response rate was 28% and the PFS was 11.2 months vs. 4 months for the placebo group [51]. For DTCs, the response prevalence was 15%, with a significant improvement in PFS compared to placebo, achieving in the last results 11.0 months [52,53]. The most common side effects reported in both clinical trials were diarrhoea, skin reaction, fatigue, and hypertension.

Other MKIs have been developed, however, they did not obtain FDA or EMA approval for TC treatment. Selumetinib received Orphan Drug Designation for patients with advanced stages of DTC. It was described that Selumetinib significantly enhances iodine uptake and retention in a subset of patients with radioiodine-refractory thyroid cancer, with potentially greater effectiveness in those with *RAS*-mutant disease [54]. This event has been reported in some MKIs previously mentioned. Vemurafenib reactivates RAI uptake and improves its effectiveness in some *BRAF*-mutant RAIR patients, possibly by increasing thyroid-specific gene activity through MAPK-pathway suppression. Elevated initial thyroglobulin levels in responders indicate that tumour differentiation may influence the likelihood of benefiting from Vemurafenib [55].

Despite encouraging data and early therapeutic success, when targeting signalling pathways with TKIs, most patients eventually experience disease progression. VEGFR inhibitors like Sorafenib and Lenvatinib continue to play a crucial role in treating DTC. However, responses are frequently partial, and long-term side effects present a substantial challenge. Resistance arises due to the activation of alternative signalling pathways, such as FGF2, PI3K/AKT, and JAK-STAT [56].

### 3.2. Combined Targeted Therapies

**Dabrafenib and trametinib:** The FDA has approved this combined therapy for *BRAF*-mutant ATC management. Also, the unique use of Dabrafenib (or combined with trametinib) obtained the approval of the FDA for RAIR DTC with *BRAF* mutations.

Dabrafenib is a selective ATP–competitive inhibitor of BRAF kinases, and it has been probed in cell lines and xenografts. This inhibitor targets the *BRAF* V600E mutation, reducing ERK phosphorylation and suppressing cell proliferation. It initially causes cell cycle arrest in the G1 phase, followed by apoptosis. This effect is selective for cancer cells harbouring the *BRAF* V600E mutation [57]. Dabrafenib is associated with a decrease in phosphorylated ERK levels. A clinical trial demonstrated a good response in metastatic tumours, including PTCs. Nevertheless, most patients acquired resistance, activating the downstream MAPK pathway [58]. Thus, this has entailed new clinical trials with the combination of dabrafenib and trametinib (Table 1).

Trametinib is a selective MEK inhibitor that targets MEK1 and MEK2 in an ATP non-competitive manner [59]. In vitro assays demonstrate that the combination of dabrafenib and trametinib is associated with suppressed cell growth, reduced ERK phosphorylation, decreased cyclin D1 protein expression, and elevated p27 protein levels in cell lines exhibiting acquired resistance to dabrafenib monotherapy [60]. The clinical trial ROAR corroborates previous studies that show the combination of dabrafenib and trametinib exhibits significant clinical activity in advanced or metastatic ATC harbouring the *BRAF* V600E mutation. It reported a response rate of 56% and a PFS of 6.7 months. The most prevalent side effects described were pyrexia, anaemia, decreased appetite, and fatigue [61].

The clinical trial studying the use of monotherapy and combination therapy with these new anti-cancer drugs found that RAIR-DTC tumours had a 35% response rate with dabrafenib alone, compared to a 30% response rate when dabrafenib was combined with trametinib. PFS was 10.7 months with dabrafenib and 15.1 months with the combined drugs. The most common adverse events described were skin disorders and fever [62]. Recent results showed that combined dabrafenib and trametinib therapy was effective in *BRAF* V600E-mutated DTC patients for restoring 131I uptake in 38% of patients [63].

The redifferentiation effect reported for MKIs has also been demonstrated for dabrafenib in *BRAF* V600E RAIR-PTCs [64]. The combination of dabrafenib and trametinib demonstrated in patients with *BRAF* V600E mutations has proven effective in reactivating RAI uptake. This treatment leads to tumour control in 90% of patients and tumour response in 38%, with minimal side effects [65].

**Vemurafenib and Cobimetinib:** Vemurafenib is a *BRAF* selective inhibitor that targets the ATP binding site of the *BRAF* V600E mutation. The clinical trial in RAIR DTC reported a 38.5% response rate and a PFS of 18.2 months. Common side effects were reported (rash, fatigue, or weight loss) (Table 1) [66]. The major problem of Vemurafenib remains in the development of resistance after several months of treatment in TC; however, use with other MAPK inhibitors such as Cobimetinib (an MEK inhibitor) demonstrated a promising perspective [67,68]. The DETERMINE clinical trial is now taking place for *BRAF*-mutant PTC patients [69]. Mutation-guided MAPK-pathway inhibition—when using this combination of anti-cancer drugs under concurrent thyroid hormone withdrawal—represents a feasible and promising approach for redifferentiating RAIR DTC. This strategy enhances their responsiveness to RAI therapy, ensuring effective retention following treatment [70].

**Vemurafenib and SPH2 inhibitors**: SHP2 is a key target protein in the RTK signalling pathway. Preclinical assays showed that its reduction, either through SHP2 knockdown or inhibition with SHP099, significantly enhances early sensitivity to Vemurafenib and reverses late resistance in *BRAF* V600E-mutant TC cells [71].

Resistance to BRAF inhibitors in TC exhibits tissue-dependent variability in its response. Evidence suggests that inhibition of a single RTK may be insufficient to overcome primary resistance to MAPK-pathway inhibition in BRAF-mutant tumours [72]. In PTC, a resistance mechanism has been identified involving the activation of HER2 and HER3 receptors by neuregulin-1 (NRG1). This activation subsequently stimulates the RAS-RAF-MEK-ERK and PI3K-AKT signalling pathways, contributing to tumour progression [73].

Furthermore, in TC, the activation of the transcription factor YAP has been shown to create a dependency on this protein for tumour cell survival. YAP regulates adaptive resistance to RAF kinase inhibitors by inducing a gene expression program in *BRAF* V600E-mutant cells, which includes components of the NRG1 signalling pathway. This pathway plays a pivotal role in the lineage-dependent insensitivity to MAPK inhibitors. Studies have demonstrated that activated YAP regulates the expression of key upstream components of the NRG1 pathway, such as NRG1, HER3, and HER2. Notably, mutations in the HIPPO pathway involving YAP are infrequent in TC. Consequently, YAP and TEAD activity may function as a critical regulator, modulating the extent of adaptive changes in RTK signalling following MAPK-pathway inhibition in *BRAF* V600E-mutant cancers [74]. A combination of therapies such as the previously mentioned could be a good option to tackle TC treatment resistance.

### 3.3. RET Selective Inhibitors

Despite their clinical benefits, MKIs have high toxicity and limited efficacy, especially against *RET* V804 gatekeeper mutations [75]. Selective RET inhibitors (RETis) were developed for *RET*-altered cancers, including MTC, where *RET* is the primary driver mutation [76]. RETis include selpercatinib, approved by both the EMA and FDA in 2020, and pralsetinib, FDA-approved in 2020 (Table 1) [77]. Both selective drugs showed a similar tumour size reduction, though selpercatinib tended to achieve more complete responses. Higher response rates were reported in *RET* fusion-positive cancers (79–89%) than in *RET*-mutated cases, and they were more effective against *RET* V804 mutations in MKI-pretreated patients [78].

**Selpercatinib** is an ATP-competitive selective RET kinase inhibitor [79]. Preclinical studies demonstrated nanomolar effectivity against diverse *RET* alterations, including V804 resistance mutations, with strong anti-tumour activity, even in brain metastases [80]. This high degree of selectivity is maintained against critical anti-targets in cells, including KDR (VEGFR2) [81]. However, RETi resistance can emerge through solvent-front mutations in *RET*, which alter the RETi binding affinity [82]. The clinical trial LIBRETTO showed high efficacy in RET-altered TCs. The response rate ranged from 69 to 79% with 1-year PFS rates of 64–92%, benefitting both treatment-naïve and previously treated patients [83]. In LIBRETTO-531, selpercatinib demonstrated superior efficacy over MKIs (Vandetanib and Cabozantinib) in naïve MTC patients. It achieved a 69% response rate and 86% 1-year PFS, nearly doubling MKI outcomes. This significant improvement highlights selpercatinib’s potential as a breakthrough therapy for MTC. Common adverse events include hypertension and increased alanine and aspartate aminotransferase levels [84].

**Pralsetinib** is a highly selective RET kinase inhibitor with sustained anti-tumour activity. Preclinical studies showed potent efficacy against various *RET* fusions and gatekeeper mutations (V804L/M/E), with superior selectivity over MKIs. Unlike MKIs, pralsetinib inhibited RET-driven tumour growth without VEGFR2 inhibition, potentially reducing off-target toxicities [85]. Furthermore, certain solvent-front *RET* mutations—such as p.G810C/S/R, p.Y806C/N, and p.V738A—can reduce pralsetinib’s effectiveness by interfering with the binding site [86]. In the ARROW trial, *RET*-mutant patients achieved a 60–71% response rate and 75–81% 1-year PFS. *RET* fusion-positive patients showed the highest response, with an 89% response and 81% PFS [87]. These results were later confirmed in subsequent trials. Pralsetinib was associated with a high incidence of treatment-related adverse events (97.1%). The most common were elevated aspartate aminotransferase levels, anaemia, and hypertension [88].

Several selective RETis are currently in early clinical and preclinical development. These drugs aim to overcome solvent-front mutations that limit the effectiveness of approved RETis and reduce associated adverse effects [89].

**Zeteletinib** has demonstrated strong anti-tumour activity in *RET*-altered tumours, with a 44% response rate in MTC patients. This RETi has shown a favourable safety profile, with no significant hepatotoxicity or hypertension observed. It has also exhibited dose-dependent exposure and an extended half-life [90].

**Vepafestinib** demonstrated improved selectivity and more potent inhibition of *RET*-WT and *RET* G810 solvent-front mutations in vitro compared to first-generation inhibitors. X-ray crystal structures of the complex revealed that it has a unique binding mode to RET concerning selpercatinib and pralsetinib: it does not fill the space in the direction of the side chain of G810, suggesting that it effectively circumvents steric hindrance from the solvent-front mutations [91]. Preclinical data suggested that vepafestinib achieves superior central nervous system penetration compared to other RET inhibitors, indicating its potential for treating brain metastases [92].

**SY5007** has shown promising results, with a 62% response rate and a 94% disease control rate in RET-altered tumours, including MTC. The drug demonstrated durable tumour regression, with rapid absorption and dose-dependent increased exposure [93]. Similarly, **EP0031** exhibited efficacy in *RET*-altered patients in Phase I/II clinical trials. Notably, five out of six patients with central nervous system metastases experienced an intracranial response, highlighting the need for further investigation into EP0031 as a promising approach for treating brain metastases in TC [94].

### 3.4. RAS Inhibitors

**Farnesyltransferase (FTase) inhibitors:** FTase, a zinc metalloenzyme, has a pivotal role in the RAS pathway, which is essential for the post-translational modification of RAS. FTase attaches 15-carbon isoprenoids to RAS proteins, crucial for membrane-attaching in RAS activation [95]. The most well-known FTase inhibitor, tipifarnib, attaches a farnesyl isoprenoid lipid to the cysteine of the CAAX box of the RAS C-terminal. FTase inhibitors are more active against HRAS compared to NRAS or KRAS in cancer cell lines [96]. Untch et al. demonstrated that tipifarnib produced noticeable responses and improved survival in mice with HRAS-driven TC. It primarily stabilised the disease or reduced tumour growth in most instances. As noted, HRAS delocalisation correlated with the activation of WT RAS and adaptive drug resistance, which could be countered in vitro by inhibiting specific upstream RTKs that drive WT RAS activation. Treatment of HRAS-mutant tumours with tipifarnib over long periods resulted in resistant tumours that harbour a mutation in *NF1* and *GNAS* [97]. This therapy combined with Sorafenib was tested in a clinical trial, showing safety and excellent tolerance in TC above all those with *RET* mutations [98]. It also has been combined with CDK4/6 inhibitors in ATC cell lines. This preclinical assay suggests its potential use for specific molecular subgroups of advanced TC [99].

Novel **natural compounds** from *Achyranthes aspera Linn* were discovered to target BRAF and NRAS in in silico analyses. These results suggest that natural plant agents could be an excellent source of potential anti-cancer agents for TC. Seven possible *Achyranthes aspera* compounds passed the drug-likeness rule and exhibited less toxicity than the co-crystallised inhibitors. The active site residues form substantial intramolecular interactions, including hydrogen bonds and hydrophobic forces, which help stabilise the protein–ligand complex [100]. More preclinical analyses are needed to determine the anti-tumourigenic effect of these putative drugs and their side effects.

### 3.5. Other Potential Selective Inhibitors for TC

***NTRK*** fusion-positive TC is targeted by two new treatments, entrectinib and larotrectinib, with a response rate of 53.8% and 71%, respectively [101]. Tumour models with *NTRK* mutations treated with these agents resulted in the inhibition of the MAPK, PI3K–AKT, PKC, and STAT3 pathways. However, solvent-front mutations, derived from acquired resistances, have been reported in the literature [102].

Several new anti-cancer drugs were developed to target ***TERT***. The inhibition of TERT could signify a new approach in TC due to the aggressivity that confers to the tumours that harboured mutations in its promoters or other activation events.

BIBR1532 has been suggested as a treatment for ATCs. Preclinical studies demonstrated its capacity to destabilise the telomere structure while tumour cells initiate apoptosis, increasing their sensitivity to replicative senescence. Turkmen et al. showed in ATC cells that BIBR1532 caused changes in the expression of apoptotic genes in the extrinsic and intrinsic pathways [103]. Improvements in this drug are still being realised. Modifications based on substituting the terminal phenyl lipophilic head with a para chloro-group achieved the best telomerase inhibition [104].

Imetelstat is another compound proposed for future *TERT*-mutant cancers. Imetelstat binds to a complementary 13-nucleotide region of the telomerase RNA, displaying strong affinity and specificity at the active dominium of the telomerase holoenzyme, inhibiting its activity. Its effectiveness has been probed in several cancer cell lines and xenografts [105]. This compound received FDA approval in 2024 for the treatment of myelodysplastic syndromes, marking a significant milestone as the first-ever telomerase inhibitor to be approved by the FDA. This groundbreaking approval represents a major advancement in cancer treatment.

Other selective inhibitors that target *TERT* include the novel small molecule, G-quadruplex. Small-molecule ligands can limit cancer telomere lengthening by targeting telomere G4 (a guanine-rich sequence of the telomeric DNA) and TERT [106]. Long et al. demonstrated in preclinical assays of triple-negative breast cancer cell lines and xenografts that these ligands downregulate TERT expression through mitochondrial dysfunction, disrupting iron metabolism and activating ferroptosis [107].

A new compound has been reported that could be effective in ***EIF1AX*** TC tumours. CM16 decreases the translation of neosynthesised proteins in vitro while not affecting mRNA transcription. CM16 rapidly penetrates the cell and targets translation initiation, highlighted by ribosomal disorganisation. CM16 did not induce cell cycle arrest but it is cytostatic. This drug emerged as a promising alternative for *EIF1AX*-mutant TC [108].

### 3.6. Immunotherapies

Although immunotherapies have been a new paradigm in several cancer treatments, TC trials are still ongoing today. The TC microenvironment is characterised by the presence of a diverse spectrum of immune cells. While certain immune cell populations may contribute to tumour suppression, many promote inflammatory and tumourigenic processes, such as cytokines and chemokines. This process of tumour-associated immune activation is marked by an increase in T cells and regulatory T cells expressing immune checkpoints—such as programmed cell death protein 1 (PD-1)—which have been associated with more aggressive disease phenotypes, including extranodal invasion. Furthermore, elevated programmed cell death ligand 1 (PD-L1) expression has correlated with disease progression. Several new anti-cancer drugs have been focused on targeting PD-L1 [101].

**Pembrolizumab:** This is a humanised, selective immunoglobulin G4/κ anti-PD-1 monoclonal antibody with anti-tumour activity by blocking the interaction between PD-1 and its ligands. Phase I of the clinical trial KEYNOTE has shown pembrolizumab’s safety, tolerability, and anti-tumour activity in patients with PD-L1-positive, advanced DTC [109]. In Phase II, patients with advanced tumours were enrolled regardless of PD-L1 status. The response rate was 6.8% and the PFS was 4.2 months. Side effects were observed in almost 70% of patients (mainly, fatigue, pruritus, and rash) (Table 1) [110]. The combination of Lenvatinib and pembrolizumab has been shown to be safe and effective in patients with ATC or PDTC and can result in complete and long-term remissions [111]; this has also been shown in RAIR-DTC patients [112]. Hamidi et al. also reported the addition of pembrolizumab to dabrafenib/trametinib may significantly prolong survival in *BRAF* V600E-mutant ATCs [113]. 

**Spartalizumab:** This immunotherapy is also an immunoglobulin G4κ monoclonal antibody that binds PD-1 with subnanomolar activity in vitro and blocks the interaction with PD-L1 [114]. This treatment was tested in ATCs (Table 1). The overall response was 19%, with 1-year survival in 52.1% of PD-L1-positive patients [115].

**Atezolizumab:** This anti-cancer drug is an effector-less (FcγR-binding deficient) humanised immunoglobulin G1 monoclonal antibody that targets PD-L1 and blocks its interaction with PD-1 and B7.1 [116]. A specific characteristic of atezolizumab is its deficiency in FcγR binding due to an asparagine-to-alanine substitution at position 298 within the CH2 domain of each heavy chain. Consequently, it is unable to bind to Fc receptors on phagocytes and does not induce antibody-dependent cell-mediated cytotoxicity [117]. A non-randomised clinical trial demonstrated that mutation-directed targeted therapy combined with PD-L1-inhibitor immunotherapy represents a promising approach (atezolizumab + Vemurafenib/Cobimetinib) for prolonging overall survival in patients with ATC. The study outcome was to achieve 19 months of survival in the complete targeted therapy cohort, compared to the historical data of 5 months [118].

Several combinations of immunotherapies are currently being developed and studied in TC clinical trials. Nivolumab/Ipilimumab + Cabozantinib has been demonstrated as a promising therapeutic option in advanced and metastatic DTC, which have been previously treated with anti-VEGFR drugs. In ATCs, Durvalumab + tremelimumab+ radiation has improved survival in metastatic ATCs with no other therapeutic option [101].

## 4. Conclusions

Numerous new treatments are now under development, with different targets across the spectrum of TC alterations. Preclinical assays and early stages of clinical trials give the scientific community a promising perspective for the future of TC patients.

Personalised medicine has been a milestone in disease management, allowing new therapeutic opportunities for patients with the most aggressive TC histotypes to emerge. The genomic analyses and the knowledge and significance of molecular alterations have allowed us to increase the PFS of all TC histotypes during the last few years. Currently, the most relevant genetic markers used by clinicians for predicting tumour behaviour are TC drivers (*BRAF, RAS,* or RTK, such as *RET* or *NTRK* alterations). *TERT*-promoter mutations have been established as indicative of aggressivity.

Clinical trials investigating DTC subtypes have reported heterogeneous outcomes, with Lenvatinib and Cabozantinib demonstrating superior PFS and hazard ratios (HRs) for FTC compared to PTC; Sorafenib indicated the poorest HR for oncocytic variants; and pembrolizumab exhibited higher disease progression rates in FTC than in PTC [41,45,52,109]. Overall, no consistent response pattern has been established, although oncocytic thyroid carcinoma is associated with a poorer prognosis, increased metastatic and recurrence risk, and reduced sensitivity to radioactive iodine therapy compared to FTC [119].

New anti-cancer drugs mentioned in this review and others such as nanoparticles—a new technology evolving with high activity, less toxicity, and sustained release to target tissue [120]—could continue to improve TC patients’ prognoses. Anti-diabetic compounds have also been suggested as new agents against TC [121]. More studies about these putative cancer agents are needed to determine their potential clinical use.

Novel therapeutic agents exhibit improved tolerability compared to chemotherapies. Furthermore, resistance to these inhibitors presents a significant challenge that must be tackled. A comprehensive investigation will be essential to elucidate the underlying mechanisms of resistance and to develop effective targeted treatment approaches.

Nevertheless, combined therapies have emerged as a promising advancement to enhance efficacy and overcome resistance to single-agent TC anti-cancer drugs. Immunotherapy and selective inhibitors have been demonstrated to be auspicious options for TC patients. Despite these approaches, challenges arise, including adverse events management and identifying the most effective combinations for specific patient subgroups. Ongoing clinical trials aim to improve outcomes and treatment options for patients with advanced and aggressive TC histotypes.

## Figures and Tables

**Table 1:** Detailed information on the most relevant clinical trials for thyroid cancer. PFS: progression-free survival, DTC: differentiated thyroid cancer, MTC: medullary thyroid cancer, ATC: anaplastic thyroid cancer, TC: thyroid cancer.

Drug	Target	Indication	Clinical Trial	Efficacy		Common Adverse Effects
				PFS	Response Rate	
Sorafenib	VEGFR-1, VEGFR-2, VEGFR-3, RET, BRAF, KIT, and PDGFR	RAIR DTC	DECISION Phase III	10.8 months	12.2%	Secondary malignancy, dyspnoea, and pleural effusion
Lenvatinib	VEGFR-1, VEGFR-2, VEGFR-3, RET, BRAF, KIT, PDGFR, and FGFR	RAIR DTC	SELECT Phase III	18.3 months	64.8%	Diarrhoea, hypertension, proteinuria, and decreased appetite
Vandetanib	VEGFR, PDGFR, EGFR, and RET	MTC	NCT00410761 Phase III	30.5 months	45%	Diarrhoea, rash, nausea, hypertension, and headache
Cabozantinib	MET, RET, KIT, and VEGFR	MTC	EXAM Phase III	11.2 months	28%	Diarrhoea, skin reaction, fatigue, and hypertension
		Second-line RAIR DTC	Cosmic-311 Phase III	11 months	15%	
Dabrafenib	*BRAF* V600E mutation	RAIR DTC with *BRAF* mutations	NCT01723202 Phase II	10.7 months	35%	Skin and subcutaneous tissue disorders, fever, and hyperglycemia
Dabrafenib + trametinib	*BRAF* V600E mutation + MEK1 and MEK2	BRAF-mutant ATC	ROAR Phase II	6.7 months	56%	Pyrexia, anaemia, decreased appetite, and fatigue
		RAIR DTC with *BRAF* mutations	NCT01723202 Phase II	15.1 months	30%	Fever, nausea, chills, and fatigue
Vemurafenib	BRAF	RAIR DTC	NCT01286753 Phase II	18.2 months	38.5%	Rash, fatigue, or weight loss
Selpercatinib	RET	RET-altered TC	LIBRETTO Phase I/II	1-year PFS rate 64–92%	69–79%	Hypertension and increased alanine and aspartate aminotransferase levels
		RET-altered MTC	LIBRETTO-531 Phase III	1-year PFS rate 86.8%	69.4%	
Pralsetinib	RET	*RET*-mutant MTC	ARROW Phase I/II	1-year PFS rate 75–81%	60–71%	Elevated aspartate aminotransferase, anaemia, and hypertension
		*RET* fusion-positive TC	ARROW Phase I/II	1-year PFS rate 81%	89%	
Pembrolizumab	PD-1	Papillary or follicular TC	KEYNOTE-158 Phase II	4.2 months	6.8%	Fatigue, pruritus, and rash
Spartalizumab	PD-1	ATC	NCT02404441 Phase I/II	1-year PFS 52.1% of PD-L1 + patients	19%	Diarrhea, pruritus, fatigue, and pyrexia

## Data Availability

No new data were created or analysed in this study. Data sharing does not apply to this article.

## Abbreviations

The following abbreviations are used in this manuscript:

ALK	Anaplastic lymphoma kinase
ATC	Anaplastic thyroid carcinoma
ATP	Adenosine triphosphate
CDK	Cyclin-dependent kinases
cMYC	Cardiac myosin-binding protein C
DHGTC	Differentiated high-grade thyroid carcinoma
DTC	Differentiated thyroid carcinoma
EGFR	Epidermal growth factor receptor
EIF1AX	Eukaryotic translation initiation factor 1A X-linked
EMA	European Medicines Agency
ERK	Extracellular signal-regulated kinase
FDA	Food and Drug Administration
FGFR	Fibroblast growth factor receptor
FTase	Farnesyltransferase
HGF	Hepatocyte growth factor
HR	Hazard ratio
MEN2A/2B	Multiple endocrine neoplasia type 2A/2B
MEK	Mitogen-activated extracellular signal-regulated kinase
MKI	Multikinase inhibitor
MAPK	Mitogen-Activated Protein Kinase
MTC	Medullary thyroid carcinoma
NF1	Neurofibromin
NIS	Sodium Iodide Symporter
NRG1	Neuregulin 1
NTRK	Neurotrophic tropomyosin-receptor kinase
PDGFR -β	Platelet-derived growth factor receptor-beta
PD-L1	Programmed cell death ligand 1
PD-1	Programmed cell death protein 1
PDTC	Poorly differentiated thyroid carcinoma
PFS	Progression-free survival
PIC	Preinitiation complex
PI3K	Phosphoinositide 3-kinase
PTC	Papillary thyroid carcinoma
RAIR	Radioactive iodine refractory
RET	REarranged during Transfection
RETi	RET inhibitor
RTK	Receptor tyrosine kinase
sMTC	Sporadic medullary thyroid carcinoma
TC	Thyroid cancer
TCGA	The Cancer Genome Atlas
TERT	Telomerase reverse transcriptase
VEGFR	Vascular endothelial growth factor receptor
WT	Wild type
